# Beyond the Hip: Clinical Phenotypes of Hip Osteoarthritis Across the Biopsychosocial Spectrum

**DOI:** 10.3390/jcm13226824

**Published:** 2024-11-13

**Authors:** Abner Sergooris, Jonas Verbrugghe, Bruno Bonnechère, Sim Klaps, Thomas Matheve, Frans-Jozef Vandeputte, Kristoff Corten, Katleen Bogaerts, Annick Timmermans

**Affiliations:** 1 REVAL Rehabilitation Research, Faculty of Rehabilitation Sciences, Hasselt University, 3590 Diepenbeek, Belgiumsim.klaps@uhasselt.be (S.K.); thomas.matheve@ugent.be (T.M.); katleen.bogaerts@uhasselt.be (K.B.);; 2 Technology-Supported and Data-Driven Rehabilitation, Data Sciences Institute, Hasselt University, 3590 Diepenbeek, Belgium; 3 Department of PXL—Healthcare, PXL University of Applied Sciences and Arts, 3500 Hasselt, Belgium; 4 Spine, Head and Pain Research Unit Ghent, Department of Rehabilitation Sciences, Ghent University, 9000 Ghent, Belgium; 5 Department of Orthopaedics—Hip Unit, Ziekenhuis Oost-Limburg, 3600 Genk, Belgium; 6 Centre for Translational Psychological Research (TRACE), Ziekenhuis Oost-Limburg, 3600 Genk, Belgium; 7 Department Health Psychology, Faculty of Psychology and Educational Sciences, Catholic University of Leuven, 3000 Leuven, Belgium

**Keywords:** clinical phenotypes, hip osteoarthritis, total hip arthroplasty, cognitions, emotions, trauma, quantitative sensory testing, biopsychosocial

## Abstract

**Background/Objectives**: To identify clinical phenotypes of hip osteoarthritis (OA) within a biopsychosocial framework. **Methods**: A cross-sectional analysis of 143 individuals with hip OA awaiting total hip arthroplasty (THA) was performed. Phenotyping features included sociodemographic and biomedical information, pain-related cognitions and emotions, mental disorders, traumatic experiences, self-efficacy, social support, perceived stress, and somatosensory function. Outcome measures included the hip disability and osteoarthritis outcome score and the numeric pain-rating scale. Decision tree learning was used to select the most important phenotyping features. K-means clustering analyses were performed to identify clinical phenotypes and a decision tree algorithm was trained to classify individuals in the identified clinical phenotypes. **Results**: Selected phenotyping features associated with pain and disability included a combination of biomedical, psychological, and social variables. Two distinct clinical phenotypes were identified. Individuals within the maladaptive phenotype (34%) reported more comorbidities, less self-efficacy and higher levels of anxiety, depression, pain-related fear-avoidance, and feelings of injustice. No differences were found regarding social support and somatosensory function. Regarding the outcome measures, individuals within the maladaptive phenotype reported higher levels of pain and disability. Finally, based on the Fear-Avoidance Components Scale (FACS) and the anxiety subscale of the Hospital Anxiety and Depression Scale (HADS-A), individuals could be classified into the clinical phenotypes with 87.8% accuracy. **Conclusions**: Two clinical phenotypes, an adaptive and a maladaptive phenotype, can be identified in individuals with hip OA using the FACS and HADS-A. The identification of these clinical phenotypes represents a crucial step toward precision medicine, enabling the development of targeted treatment pathways tailored to the distinct biomedical and psychological features of each phenotype.

## 1. Introduction

Osteoarthritis (OA) is one of the most prevalent musculoskeletal disorders and a major cause of pain and disability worldwide [1,2]. Originally, OA was considered a purely structural wear-and-tear disorder, characterised by progressive degeneration of articular cartilage. In recent decades, however, OA has undergone a conceptual transformation, as considerable heterogeneity exists in the clinical and structural manifestation of OA [3]. Beyond its traditional peripheral origins, other factors such as psychological and neurological factors have emerged as contributors to the pain experience and disability of individuals with OA [4]. Indeed, central mechanisms, including central nervous system sensitization, are now recognized as important contributors to the clinical presentation of individuals with OA [5,6]. Despite these insights, current management strategies are often lagging behind as they fail to take this multidimensional perspective into account. Therefore, it should come as no surprise that they only show small to moderate effectiveness [7,8]. Therefore, a growing body of research has started exploring the existence of clinical phenotypes, which are defined as subgroups within the OA population that are characterised and identified based on a set of shared clinical characteristics [9,10]. The identification of such clinical phenotypes can provide insights into the various entities and underlying causes and mechanisms that contribute to the development and progression of OA [11]. Within a broader future perspective, clinical phenotypes can inform individuals and healthcare providers on the prognosis of the disease and can provide a basis for targeted treatments for these specific subgroups. This could lead to a more informed shared decision-making process and the development of more effective treatment approaches. Testing and implementation of these phenotype-specific treatments might lead to better effect-sizes of conservative treatment, better selection of individuals eligible for total hip arthroplasty (THA), and consequently better outcomes after THA. In summary, clinical phenotypes hold promise when it comes to elucidating the complex and diverse nature of hip OA, thereby paving the way for precision medicine by informing individualised treatment approaches.

Until now, research on clinical phenotypes of OA has primarily concentrated on the population of individuals with knee OA [9,10]. Distinct phenotypes based on clinical outcomes in individuals with knee OA have been associated with pain sensitization, psychological distress, radiographic severity, body mass index (BMI), muscle strength, inflammation, and comorbidities [12]. Clear differences exist in the aetiology, prognosis, and clinical presentation of individuals with knee and hip OA [13]. Nevertheless, limited evidence is available for clinical phenotypes of hip OA, and it tends to focus on a single aspect of the biopsychosocial framework [14,15]. In individuals with hip OA currently no studies have been conducted that attempt to identify clinical phenotypes based on characteristics across all domains of the biopsychosocial spectrum. However, variables such as maladaptive pain-related cognitions, emotions, and behaviour may lead to increased pain and disability [16]. Self-efficacy can affect the threat appraisal of pain and the capacity to cope with pain [17], while childhood trauma can influence coping strategies later in life via neuroendocrine, behavioural, and central nervous system adaptations [18,19]. Evidence in different musculoskeletal conditions has demonstrated differences in pain processing between individuals with and without a history of traumatic experiences [20,21], and childhood and adulthood trauma have been associated with the onset of chronic pain [22,23]. Consequently, these variables should be considered as phenotyping characteristics in individuals with OA. Therefore, this study aimed to identify clinical phenotypes of hip OA based on easily identifiable and treatable factors within the biopsychosocial framework.

## 2. Materials and Methods

### 2.1. Study Design

This is a cross-sectional analysis of baseline data from a larger longitudinal prospective cohort study (HIPPROCLIPS-trial, ClinicalTrials.gov Identifier: NCT05265858). All information and the complete protocol have been published elsewhere [24]. Ethical approval for the study was granted by the medical ethics committees of East-Limburg Hospital and Hasselt University (B3712021000002).

### 2.2. Participants and Recruitment

Participants were recruited between May 2021 and September 2023 from a secondary care setting at East-Limburg Hospital in Genk (Belgium) and the European Hip Clinic in Westerlo (Belgium). Individuals on the waiting list for a THA due to confirmed clinical or radiographic primary hip OA were invited to participate in this study. No specific preferences were applied regarding the sex of the participants. Exclusion criteria were (1) rheumatic arthritis or other rheumatic diseases, (2) avascular necrosis or other pathological conditions explaining the symptoms, (3) neurological disorders significantly influencing the symptoms of hip OA, (4) revision THA, (5) a history of pathological fractures (e.g., osteoporosis, tumour…), and (6) other planned surgical interventions during the follow-up period (e.g., contralateral THA, total knee arthroplasty (TKA)). Written informed consent was obtained from all participants prior to their inclusion in the study.

### 2.3. Phenotyping Variables

#### 2.3.1. Sociodemographic and Biomedical Information

Using a self-reported questionnaire that was not formally validated, participants were asked to indicate their age, sex, height, body weight, smoking status, educational level, marital status, and employment status. Height and body weight were used to calculate Body Mass Index (BMI). To determine the number of comorbidities, participants were asked to specify the comorbidities they had been diagnosed with using in a list of prevalent chronic health conditions and other comorbidities. Participants also had to indicate whether they performed sports (activities that are intense enough so that one sweats at least to a slight degree from them) on a regular basis (yes/no), whether they previously received physiotherapy for their hip complaint, and the number of treatment sessions they received. Radiographic severity was assessed using the Tönnis grading scale [25] by a trained orthopaedic surgeon (FJVDP) who was blinded to the clinical data. Finally, participants were asked to indicate their pain duration (months), use of pain medication (none, seldom, most days and/or nights, all days and/or nights), number of painful body regions (last week and last year), and the number of days with pain last week.

#### 2.3.2. Pain-Related Cognitions and Emotions

The Fear-Avoidance Components Scale (FACS) [26,27] was used to assess pain-related fear-avoidance. The FACS consists of 20 items, each rated on a six-point Likert scale from zero (‘completely disagree’) to five (‘completely agree’). The total score ranges from zero to one hundred, with higher scores reflecting greater fear-avoidance behaviour. The FACS has demonstrated strong reliability and validity in individuals with chronic musculoskeletal pain, including chronic hip pain [26].

Pain-related fear of movement and (re)injury was measured using the 17-item version of the Tampa Scale for Kinesiophobia (TSK-17) [28]. Items are rated on a four-point Likert scale from one (‘strongly disagree’) to four (‘strongly agree’). Total scores range from 17 to 68, with higher scores indicating a greater fear of movement. The TSK shows adequate measurement properties in individuals with chronic musculoskeletal pain [29,30,31,32].

Perceived injustice was assessed with the Injustice Experience Questionnaire (IEQ) [33]. The IEQ includes 12 items, with each item scored on a scale from zero (‘not at all’) to four (‘all the time’). The total score can range from zero to forty-eight, with higher scores indicating greater perceived injustice. The IEQ demonstrates adequate validity in individuals with musculoskeletal pain [33].

#### 2.3.3. Traumatic Experiences

The Childhood Trauma Questionnaire (CTQ) was used to assess whether participants experienced abuse or neglect during childhood [34,35]. The CTQ consists of 25 items that assess childhood maltreatment across five dimensions: (1) physical abuse, (2) physical neglect, (3) emotional abuse, (4) emotional neglect, and (5) sexual abuse. Each item is rated on a five-point Likert scale, from one (‘never true’) to five (‘very often true’). Total scores range from twenty-five to one hundred and twenty-five, with each subscale score ranging from five to twenty-five. The CTQ has demonstrated both validity and reliability in clinical populations [35].

The Traumatic Experiences Checklist (TEC) was used to assess participants’ history of 29 potentially traumatic events across six domains: (1) emotional abuse; (2) emotional neglect; (3) sexual harassment; (4) sexual abuse; (5) physical abuse; and (6) threat to life or bizarre punishment/intense pain. It includes eleven items related to family events, such as divorce or the loss of a significant person (36). Participants were asked to report whether they had experienced a traumatic event, the age at which it occurred, its duration, and the subjective impact of the trauma, rated on a scale from one (‘no impact’) to five (‘very severe impact’). The total score, ranging from zero to twenty-nine, and the average subjective impact score were calculated. The TEC has demonstrated good psychometric properties [36].

#### 2.3.4. Mental Disorders

The Mini International Neuropsychiatric Interview Simplified (MINI-S) based on the Diagnostic and Statistical Manual of Mental Disorders 5 (DSM-5) was used to identify psychiatric comorbidities [37]. The MINI-S is a brief, semi-structured diagnostic tool that assesses the most common psychiatric disorders outlined in the DSM-5, covering 17 major conditions. Additionally, the suicidal risk module from the MINI-S DSM-IV was administered [38].

The Hospital Anxiety and Depression Scale (HADS) was used to screen for symptoms of anxiety and depression. The HADS is a 14-item questionnaire that evaluates anxiety and depression symptoms without addressing physical complaints [39]. It includes two subscales: one for anxiety (HADS-A) and another for depression (HADS-D). Each item is scored from zero (‘not applicable’) to three (‘certainly applicable’). The total score for each subscale ranges from zero to twenty-one, with higher scores indicating greater symptoms of anxiety or depression. Both subscales have demonstrated good psychometric properties in populations with musculoskeletal pain [40].

#### 2.3.5. Self-Efficacy

The General Self-Efficacy Scale (GSES) was used to measure general self-efficacy [41]. The GSES assesses how an individual typically copes with stressors and challenging situations in life. It consists of ten items, each rated on a four-point Likert scale from one (‘not at all true’) to four (‘exactly true’). The total score ranges from ten to forty, with higher scores reflecting greater self-efficacy. The GSES has been demonstrated to be a valid and reliable tool for measuring general self-efficacy [42].

#### 2.3.6. Perceived Stress

Perceived stress was assessed using a single item from the Perceived Stress Scale (PSS): “In the last month, how often have you felt nervous and stressed?” [43]. The item is rated on a five-point Likert scale, ranging from zero (‘never’) to four (‘very often’).

#### 2.3.7. Social Support

The Groningen Orthopaedic Social Support Scale (GO-SSS) was used to measure social support through 12 items [44]. Each item is rated on a four-point Likert scale, ranging from one (‘never or rarely’) to four (‘often’), yielding a total score between zero and forty-eight. The GO-SSS has demonstrated adequate validity and reliability in individuals undergoing total hip or knee arthroplasty [44].

#### 2.3.8. Somatosensory Function

Quantitative sensory testing (QST) was used to measure peripheral and central somatosensory function [45,46]. A Peltier-based computerised thermal stimulator (TSA II; Medoc Ltd., Ramat-Ishay, Israel) was used to perform the QST measurements. The standardised QST protocol included measures of local and widespread hypo- and hyperalgesia, temporal summation of pain, and conditioned pain modulation and has been described in detail elsewhere [24].

**Thermal detection and pain thresholds** (in °C) were assessed following the protocol of the German Research Network on Neuropathic Pain (DFNS), both locally (at the most painful site of the hip) and remotely (at the volar aspect of the contralateral wrist), using a limits protocol [47].

**Temporal summation of pain** was measured at the contralateral volar forearm using a two-minute tonic heat stimulus with participant-controlled temperature [48]. Participants were exposed to a tonic heat stimulus (VAS 60 temperature, maximum 45 °C) and instructed to maintain their initial sensation for two minutes by adjusting the temperature (at a rate of 1 °C/s) using the response unit. To quantify temporal adaptation and summation of pain, the extent of temperature changes was recorded.

**Conditioned pain modulation** was assessed using a Dual-Thermode program with two heat stimuli (VAS 60 temperature, maximum 45 °C) [49]. The test stimulus was applied to the volar aspect of the contralateral forearm, once on its own before administering the conditioning stimulus (interstimulus interval 10 s) and once at the end of the conditioning heat stimulus at the volar aspect of the ipsilateral forearm. The difference in pain intensity between the test stimulus alone and the test stimulus during the conditioning phase at the contralateral forearm was calculated. Pain intensity was measured using a Visual Analog Scale (VAS) ranging from zero (‘no pain’) to one hundred (‘worst imaginable pain’).

### 2.4. Outcome Variables

Perceived disability was evaluated with the Hip disability and Osteoarthritis Outcome Score (HOOS). The HOOS is a 40-item self-reported questionnaire for evaluating symptoms and disability in persons with hip complaints [50,51]. Each item is rated on a five-point Likert scale, with higher scores indicating fewer complaints. The total HOOS score ranges from zero to one hundred, where higher scores reflect lower levels of disability. The Dutch version of the HOOS has been shown to be valid and reliable in individuals with osteoarthritis [52].

Pain intensity at the time of measurement was assessed with the Numeric Pain Rating Scale (NPRS), an 11-point scale ranging from zero (“no pain”) to ten (“worst possible pain”) [53]. The NPRS has appropriate measurement properties in patients with musculoskeletal pain [54,55].

### 2.5. Statistics

**Figure 1:**
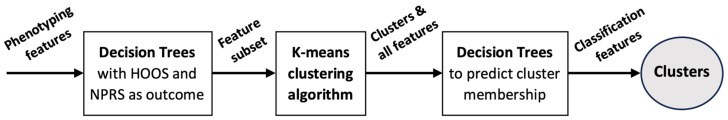
Statistical approach. Abbreviations: HOOS = Hip Disability and Osteoarthritis Outcome Score, NPRS = Numeric Pain Rating Scale.

Statistics were performed in R (Version 3.6.3, R Core Team, Vienna, Austria) using RStudio (2022.07.2, R Studio Team, Boston, MA, USA), and significance level was set at 0.05. Mean and standard deviation were used for normally distributed continuous variables, while median and interquartile range were used for non-normally distributed continuous variables. Categorical variables are presented as absolute numbers and percentages. The statistical approach to identify clinical phenotypes is presented in Figure 1. As a first step, all phenotyping features were included in a decision tree learning algorithm to predict pain and disability measured with the NPRS and the HOOS, respectively. Variable importance in the prediction was plotted, and Spearman correlation coefficients were calculated between the most important phenotyping features. If the correlation coefficient between features was higher than 0.9, one of the features was excluded from the cluster analysis. Secondly, the selected phenotyping features were included in a K-means clustering algorithm to identify clinical phenotypes. One to five-class solutions were compared and within-cluster sum of squares, average Silhouette method, and gap statistics were calculated to define the optimal number of clusters. Consequently, the identified clusters were compared on phenotyping features and outcome variables using the paired t-test or Wilcoxon signed-rank test for parametric and non-parametric continuous variables, respectively. Chi-squared tests or Fisher’s exact tests were performed to compare categorical variables. Finally, a decision tree was trained to predict cluster membership of the participants, using the selected phenotyping features as independent variables.

## 3. Results

### 3.1. Selection of Phenotyping Features

**Figure 2:**
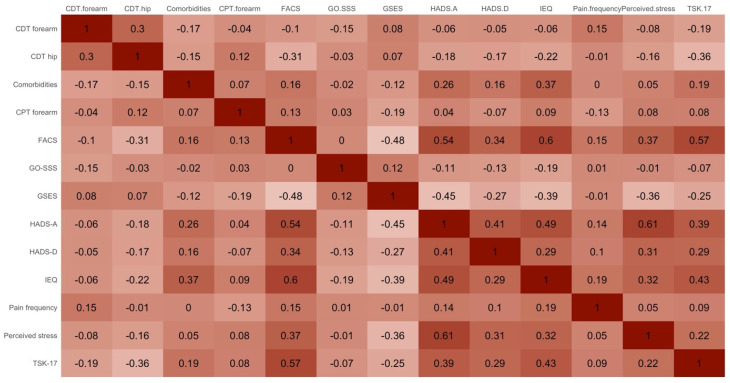
Spearman correlation coefficients between selected phenotyping features. Abbreviations: CDT = Cold detection threshold, CPT = Cold Pain Threshold, FACS = Fear-Avoidance Components Scale, GO-SSS = Groningen Orthopaedic Social Support Scale, GSES = General Self-Efficacy Scale, HADS-A = Anxiety subscale of the Hospital Anxiety and Depression Scale, HADS-D = Depression subscale of the Hospital Anxiety and Depression Scale, IEQ = Injustice Experience Questionnaire, TSK-17 = 17-item version of the Tampa Scale for Kinesiophobia.

One hundred and forty-three individuals with hip OA waiting for THA were included in the HIPPROCLIPS-trial. Variable importance plots for the decision trees trained with NPRS and HOOS as outcome measures are presented in Supplementary Figures S1 and S2. The most important features included a combination of biomedical (number of comorbidities, pain frequency, CDT and CPT at the forearm, and CPT at the hip), psychological (FACS, GSES, HADS-A, HADS-D, IEQ, TSK-17, and perceived stress), and social (GO-SSS) variables. These features were all retained for the cluster analysis, as no strong correlations were found between these features (Figure 2).

### 3.2. Identification of Clinical Phenotypes

Twenty-three (16%) of the one hundred and forty-three included individuals had missing data on one of the phenotyping features and were therefore excluded from the cluster analysis. Based on the selected phenotyping features, a two-class solution was found to best fit the data (Supplementary Figures S3–S5). As a result, two clinical phenotypes of hip OA were identified using k-means cluster analysis. The identified clinical phenotypes are described based on the phenotyping features in Table 1. Seventy-nine individuals (66%) were assigned to the first phenotype, and forty-one individuals (34%) were assigned to the second phenotype. Regarding the biological features, individuals within the second phenotype reported more comorbidities (*p* = 0.006) than individuals within the first phenotype, but no differences were found in somatosensory function. The most significant differences between both phenotypes were observed for the psychological features. Individuals within the second phenotype reported more symptoms of anxiety (*p* < 0.001) and depression (*p* < 0.001), higher levels of perceived stress (*p* < 0.001), pain-related fear-avoidance (*p* < 0.001), fear of movement (*p* < 0.001), perceived injustice (*p* < 0.001), and lower levels of self-efficacy (*p* < 0.001) compared to individuals within the first phenotype. Therefore, the second phenotype will be further referred to as the maladaptive phenotype and the first phenotype as the adaptive phenotype. Regarding social support, no differences were observed between the adaptive and the maladaptive phenotype. Finally, individuals within the maladaptive phenotype also reported higher levels of pain and disability, measured with the NPRS (*p* = 0.015) and HOOS (*p* < 0.001), respectively.

### 3.3. Prediction of Cluster Membership

**Figure 3:**
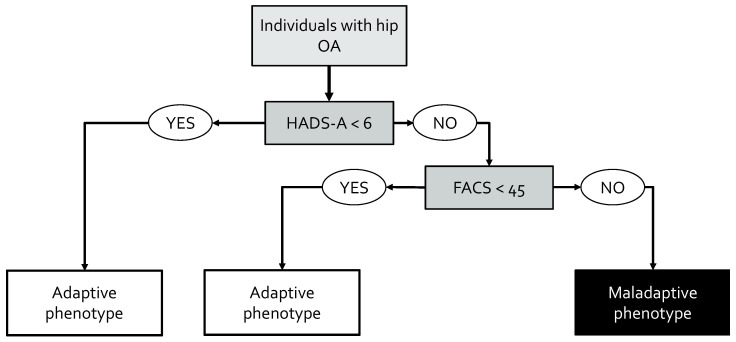
Decision tree for classification of individuals into clinical phenotypes.

Finally, a decision tree was trained to classify individuals into the clinical phenotypes (Figure 3). Individuals with (1) a score lower than six out of twenty-one on the HADS-A, or (2) a score equal or higher than six out of twenty-one on the HADS-A and a score lower than forty-five out of one hundred on the FACS, were classified within the adaptive phenotype. Other individuals were classified within the maladaptive phenotype. Accuracy of the decision tree in the test set was found to be 87.8% (95% CI = 0.738, 0.959).

## 4. Discussion

### 4.1. Summary of Findings

The aim of this study was to identify clinical phenotypes in individuals with hip OA waiting for THA. Two distinct clinical phenotypes were identified, which were defined as the adaptive and the maladaptive phenotype. Individuals within the maladaptive phenotype reported more comorbidities and appeared to have more difficulties coping with the pain and disability caused by hip OA. These individuals exhibited higher levels of maladaptive pain-related cognitions and emotions, symptoms of anxiety and depression, perceived stress, and higher levels of pain and disability. In contrast, individuals within the adaptive phenotype reported higher levels of self-efficacy and lower levels of pain and disability. However, although QST has been used to identify different underlying pain mechanisms in individuals with OA [56] and has been used for mechanistic pain profiling to predict treatment efficacy in individuals with knee OA [57], no differences were found in QST measures between the maladaptive and adaptive phenotype.

In previous research, features related to the maladaptive phenotype have been associated with poor outcomes of both conservative and surgical treatment approaches for hip OA. For example, preoperative functioning [58], comorbidities [59], and symptoms of anxiety [60] have all been associated with worse functional outcomes after THA. Further research will need to reveal whether individuals within the maladaptive phenotype are more likely to have a worse outcome after THA, as this cannot be concluded based on this cross-sectional analysis.

The findings of the current study can be explained within the conceptual framework of the fear-avoidance model of pain. The fear-avoidance model is framed within a biopsychosocial perspective and describes how individuals who experience acute pain can either get stuck in a vicious circle of pain-related fear and avoidance behaviour, leading to disuse, disability, and depression, or interpret the pain as non-threatening and follow a pattern of confrontation, leading to recovery after resuming physical activities of daily living [16]. The identified clinical phenotypes were significantly different on all these aspects of the fear-avoidance model of pain. Individuals within the maladaptive phenotype showed markedly higher scores on the FACS, a questionnaire developed to better assess all cognitive, emotional, and behavioural aspects of the fear-avoidance model, and reported higher levels of pain, disability, and depression. Furthermore, individuals in the adaptive phenotype had higher scores on the GSES, which assesses individuals’ confidence to execute a series of actions to achieve a desired outcome and cope with a stressful life situation [61]. These individuals may more easily choose the path of confrontation when experiencing pain because of their stronger self-efficacy. While our findings are consistent with the fear-avoidance model, it is important to note that this study cannot demonstrate causal relationships. Moreover, the direction and causality of these relationships remain subject to debate in the literature.

In line with previous research on clinical phenotypes of knee and hip OA, phenotypes were identified with different levels of biomedical and psychological symptoms. For example, a high distress and low distress phenotype were previously identified [62], as well as phenotypes based on different levels of central nervous system mediated symptoms [63]. Interestingly, this is the first study that explored differences between clinical phenotypes in variables across the whole biopsychosocial spectrum in individuals with OA. However, in a recent study among individuals with musculoskeletal pain, five transdiagnostic phenotypes were described based on variables across the whole biopsychosocial spectrum [64]. Our study in hip OA revealed only two phenotypes but also indicated variations in symptom levels across different biopsychosocial domains. However, apart from the differences across the biological and psychological domain, no difference was found for social support between both phenotypes. This was unexpected, as previous research has already shown that social support affects pain intensity through a direct main effect and a buffering effect on the stress response [65,66]. In addition, lower levels of social support and dissatisfaction with social support are already related to passive and maladaptive pain coping strategies [67,68] and to worse outcomes after total joint arthroplasty [69]. A more in-depth, qualitative assessment of levels and satisfaction with social support might be necessary in future studies to evaluate social support in both phenotypes.

### 4.2. Limitations

K-means clustering analysis is a widely utilised technique for partitioning data into distinct clusters based on similarity measures. However, the interpretation of results derived from this method should be approached with caution. In the present study, while both the total within-cluster sum of squares and the average silhouette method suggested a two-class solution as the optimal fit for the data, the gap statistic provided less conclusive evidence, indicating no clear benefit of a two-class solution over a one-class solution. A larger sample size might be necessary to elucidate clearer patterns and boundaries between the clinical phenotypes. Moreover, the lack of clear differentiation between the proposed clusters may suggest the existence of an “adaptive to maladaptive continuum”, rather than discrete categories. Future research should consider addressing these limitations through larger sample sizes and complementary analytical approaches.

Despite evidence in knee OA phenotypes, inflammatory, metabolic, and mechanical factors were not included as phenotyping variables in this study [10]. Instead, we prioritised selecting commonly available and easily identifiable clinical measures that span the entire biopsychosocial framework. However, we acknowledge that incorporating structural phenotypes could offer a more comprehensive view of the interactions between psychosocial factors and the physical aspects of OA. Future studies could benefit from combining structural and psychosocial phenotyping to identify subgroups that present both structural and psychosocial characteristics, thereby enhancing the precision of treatment approaches.

In addition, this study is based on cross-sectional data, which makes it impossible to validate clinical phenotypes over time. A conscious decision was made to select individuals who are eligible for a THA, ensuring that participants are at a similar clinical stage of disease. Further research is needed for internal and external validation of the clinical phenotypes and the classification algorithm.

### 4.3. Clinical Relevance

From a clinical perspective, it is important that classification of individuals within the identified clinical phenotypes can be performed with high accuracy (87.8%), using only the FACS and HADS-A in clinical practice. These questionnaires could be routinely used in clinical practice by orthopaedic surgeons, physiotherapists, and other healthcare professionals, in order to identify individuals with maladaptive pain-related cognitions and emotions, which might result in increased pain, disability, and mental disorders. The identification of these clinical phenotypes can provide a first step towards precision medicine. For example, individuals within the maladaptive phenotype might have more benefit from treatment approaches targeting their maladaptive pain-related belief, such as for example pain neuroscience education (PNE). PNE has already been shown to be effective in individuals with OA, but the effect sizes were only small for variables such as pain catastrophizing [70]. Given the characteristics of the maladaptive phenotype, effect sizes might be larger when tested specifically in individuals of this phenotype. The identification of predictors of the treatment effect of phenotype-targeted treatment approaches, and the use of single-case experimental studies will be necessary next steps to evolve from clinical phenotypes towards precision medicine.

The identified clinical phenotypes can further be an important facilitator of transdisciplinary collaboration in orthopaedic care, as these phenotypes may provide a basis for a common framework of thinking for orthopaedic surgeons, general practitioners, physical therapists, and psychologists. In this way, healthcare providers can, for example, quickly recognize and respond to maladaptive cognitions and emotions and, if necessary, facilitate referral to specialised care.

## 5. Conclusions

Two distinct clinical phenotypes were identified, referred to as the adaptive and maladaptive phenotype, which differ in features across the biological and psychological domain. Individuals with hip OA can be classified in these phenotypes using only the FACS and HADS-A questionnaire.

## Figures and Tables

**Table 1:** Comparison of clinical phenotypes on phenotyping features and outcome measures.

Variable	All Participants	Phenotype 1	Phenotype 2	*p*-Value *
*N* (%)	120 (100%)	79 (66%)	41 (34%)	/
*Comorbidities* (*n*)	1 (0,1)	0 (0, 1)	1 (0, 1)	**0.006 ^a^**
*Pain frequency* (0–7)	5 (5, 5)	5 (5, 5)	5 (5, 5)	0.875 ^a^
*HADS-A* (0–21)	4.00 (2.00, 6.25)	2.00 (1.00, 4.00)	8.00 (6.00, 11.00)	**<0.001 ^a^**
*HADS-D* (0–21)	8.00 (7.00, 10.00)	8.00 (6.00, 9.00)	10.00 (8.00, 13.00)	**<0.001 ^a^**
*FACS* (0–100)	40.83 (16.88)	33.37 (12.75)	55.20 (14.46)	**<0.001 ^b^**
*TSK-17* (17–68)	37.26 (6.37)	34.91 (5.50)	41.78 (5.47)	**<0.001 ^b^**
*GSES* (0–40)	31.00 (28.00, 35.00)	33.00 (29.50, 37.00)	28.00 (24.00, 30.00)	**<0.001 ^a^**
*Perceived stress* (0–4)	2 (2, 3)	2 (2, 3)	3 (3, 4)	**<0.001 ^a^**
*GO-SSS* (0–48)	34.50 (28.00, 40.25)	37.00 (30.00, 41.00)	32.00 (26.00, 39.00)	1.000 ^a^
*IEQ* (0–48)	5.00 (2.00, 12.00)	4.00 (1.00, 7.00)	12.00 (8.00, 18.00)	**<0.001 ^a^**
*CDT forearm*	30.71 (29.99, 31.12)	30.76 (30.20, 31.12)	30.58 (29.33, 31.10)	1.000 ^a^
*CPT forearm*	12.74 (5.18, 22.56)	11.87 (3.89, 18.91)	17.56 (9.16, 24.37)	0.557 ^a^
*CDT hip*	28.27 (26.20, 29.38)	28.57 (26.80, 29.52)	27.09 (26.01, 28.54)	0.143 ^a^
*HOOS* (0–100)	38.42 (13.86)	42.47 (13.39)	31.28 (13.00)	**<0.001 ^b^**
*NPRS* (0–10)	5.42 (2.34)	4.85 (2.21)	6.29 (2.19)	**0.015 ^b^**

Legend. Results are reported as median (Q1, Q3) or mean (SD). ^a^ Wilcoxon signed-rank test, ^b^ Independent samples *t*-test, * Bonferroni correction for multiple comparison. Abbreviations: HADS-A/D = Hospital Anxiety and Depression Scale subscale Anxiety/Depression, FACS = Fear-Avoidance Components Scale, TSK-17 = 17-item Tampa Scale for Kinesiophobia, GSES = General Self-Efficacy Scale, GO-SSS = Groningen Orthopaedic Social Support Scale, IEQ = Injustice Experience Questionnaire, CDT = Cold Detection Threshold, CPT = Cold Pain Threshold, HOOS = Hip Disability and Osteoarthritis Outcome Score, NPRS = Numeric Pain Rating Scale.

## Data Availability

The authors are not permitted to share the data used in this study due to the lack of informed consent and ethical approval for data sharing. Additionally, data sharing is not allowed for proprietary reasons.

## Supplementary Materials

The following supporting information can be downloaded at: https://www.mdpi.com/article/10.3390/jcm13226824/s1, Figure S1: Variable importance plot NPRS; Figure S2: Variable importance plot HOOS; Figure S3: Within-cluster sum of squares, Figure S4: Average Silhouette method, Figure S5: Gap statistic.
