# Supplementary figures and images for: Beyond the Hip: Clinical Phenotypes of Hip Osteoarthritis Across the Biopsychosocial Spectrum
Source: J Clin Med. 2024 Nov 13;13(22):6824. doi: 10.3390/jcm13226824 (PMC11594843; doi:10.3390/jcm13226824)

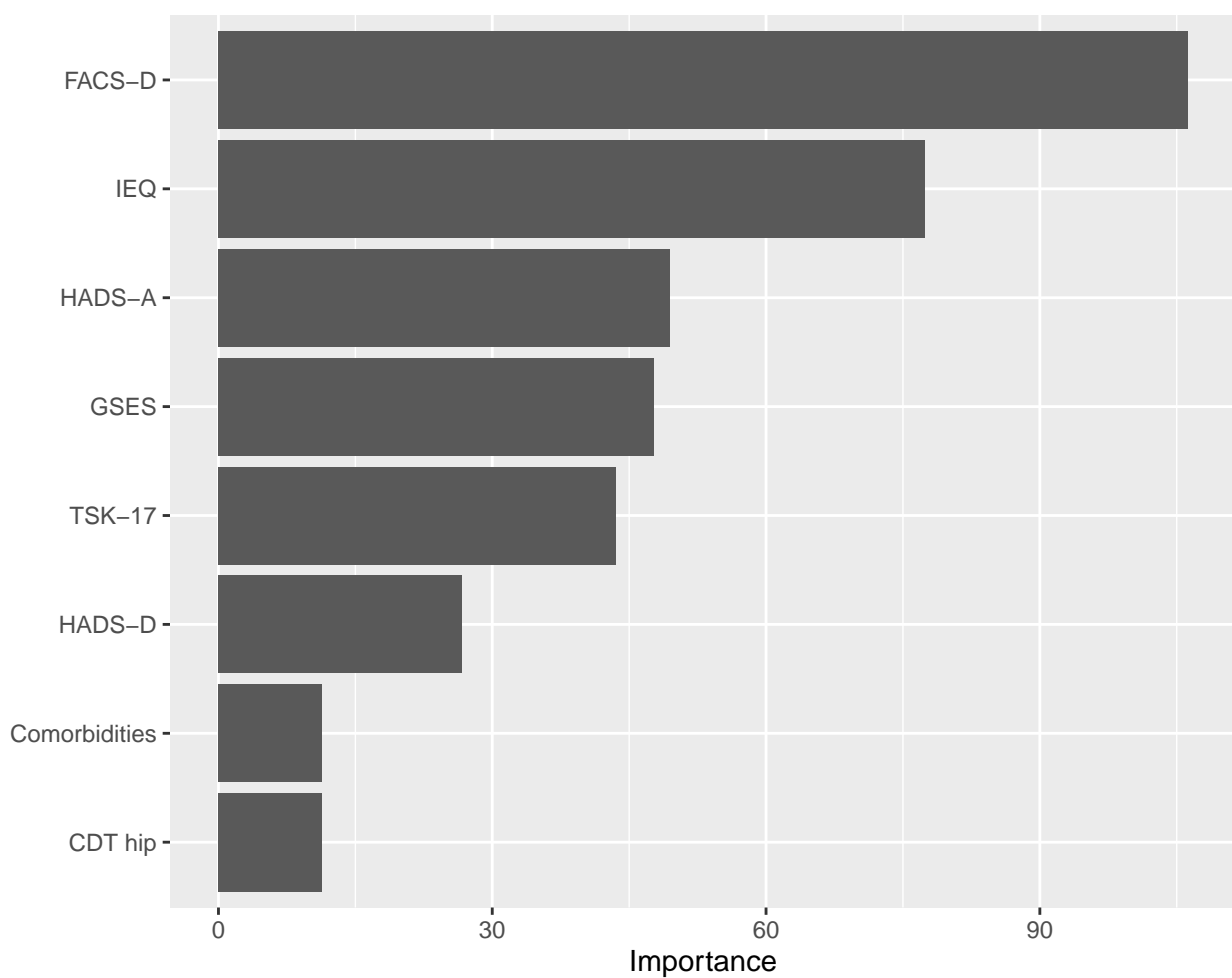

Supplement: Supplementary file 1 [file jcm-13-06824-s001.zip › Figure S1. Variable importance plot NPRS.pdf]

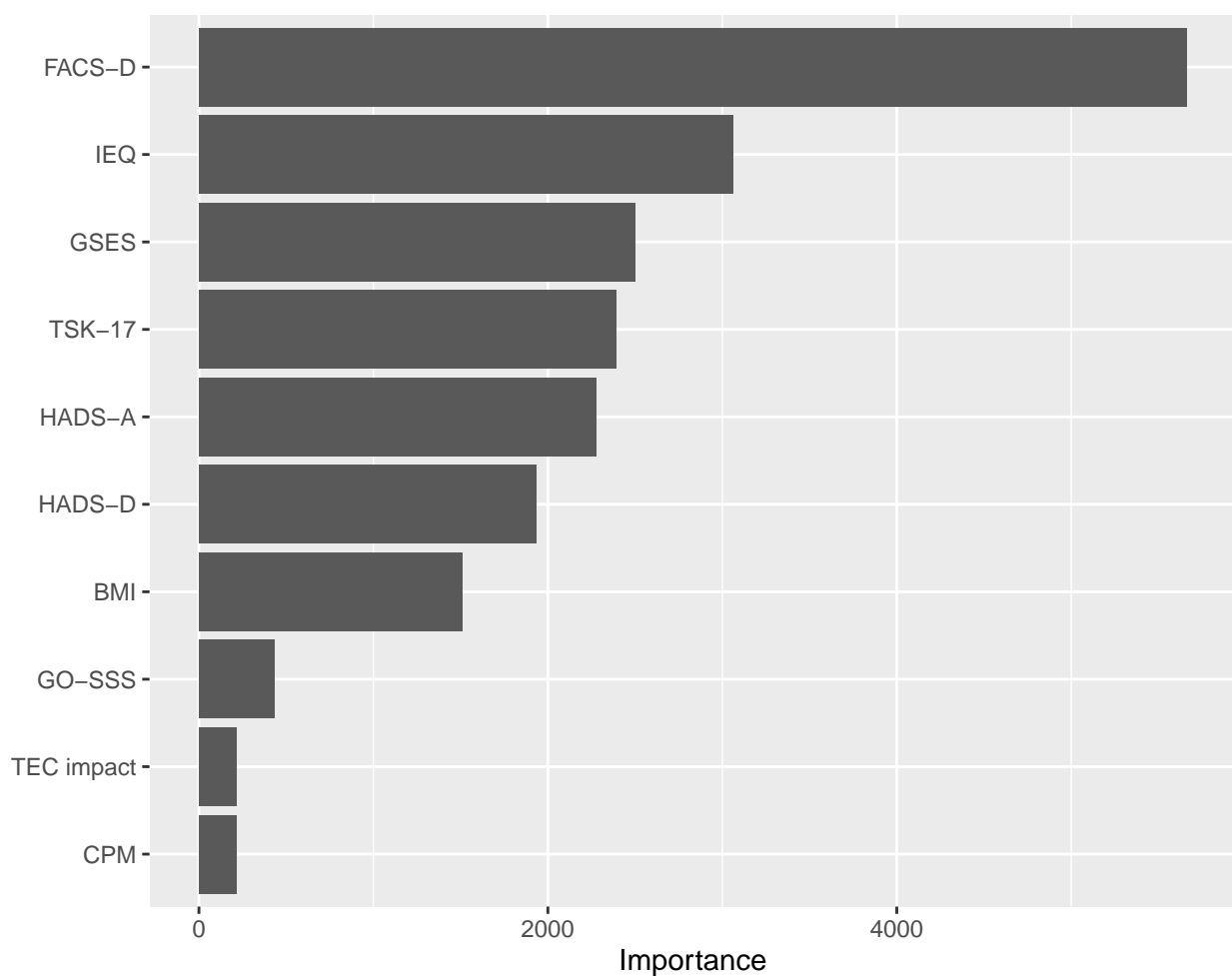

Supplement: Supplementary file 1 [file jcm-13-06824-s001.zip › Figure S2. Variable importance plot HOOS.pdf]

Optimal number of clusters

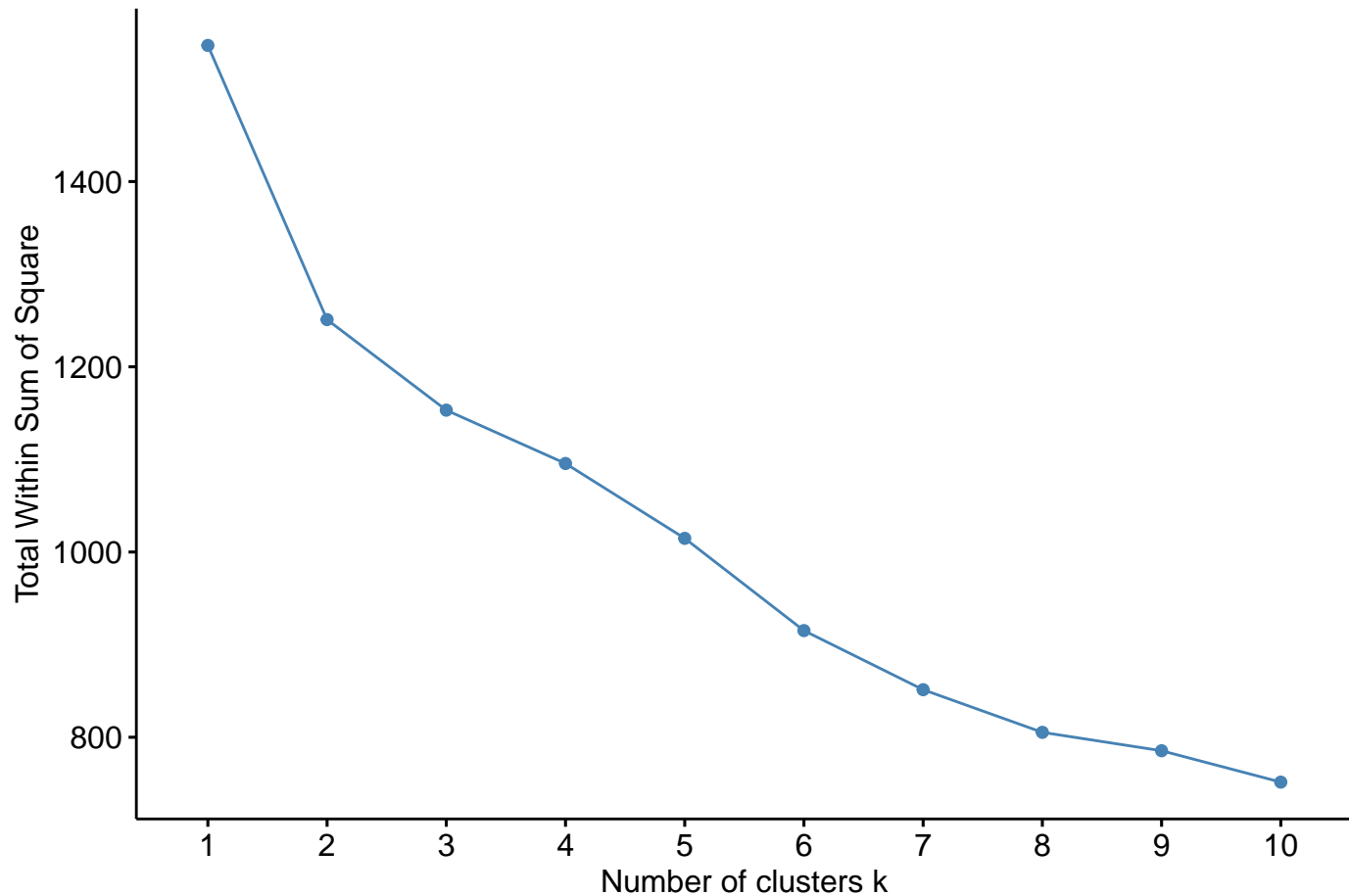

Supplement: Supplementary file 1 [file jcm-13-06824-s001.zip › Figure S3 - Within-cluster sum of squares.pdf]

Optimal number of clusters

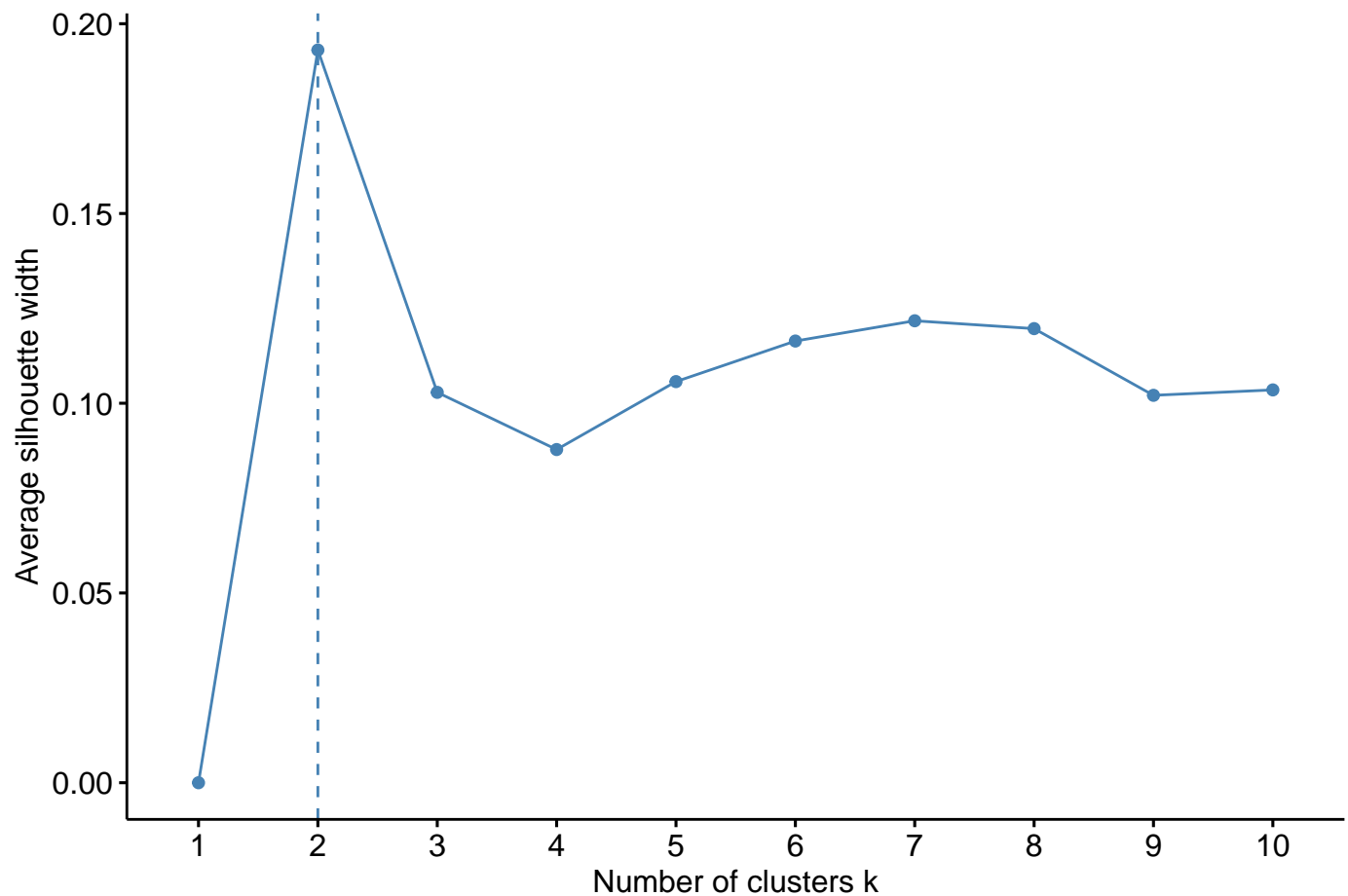

Supplement: Supplementary file 1 [file jcm-13-06824-s001.zip › Figure S4 - Average Silhouette method.pdf]

Optimal number of clusters

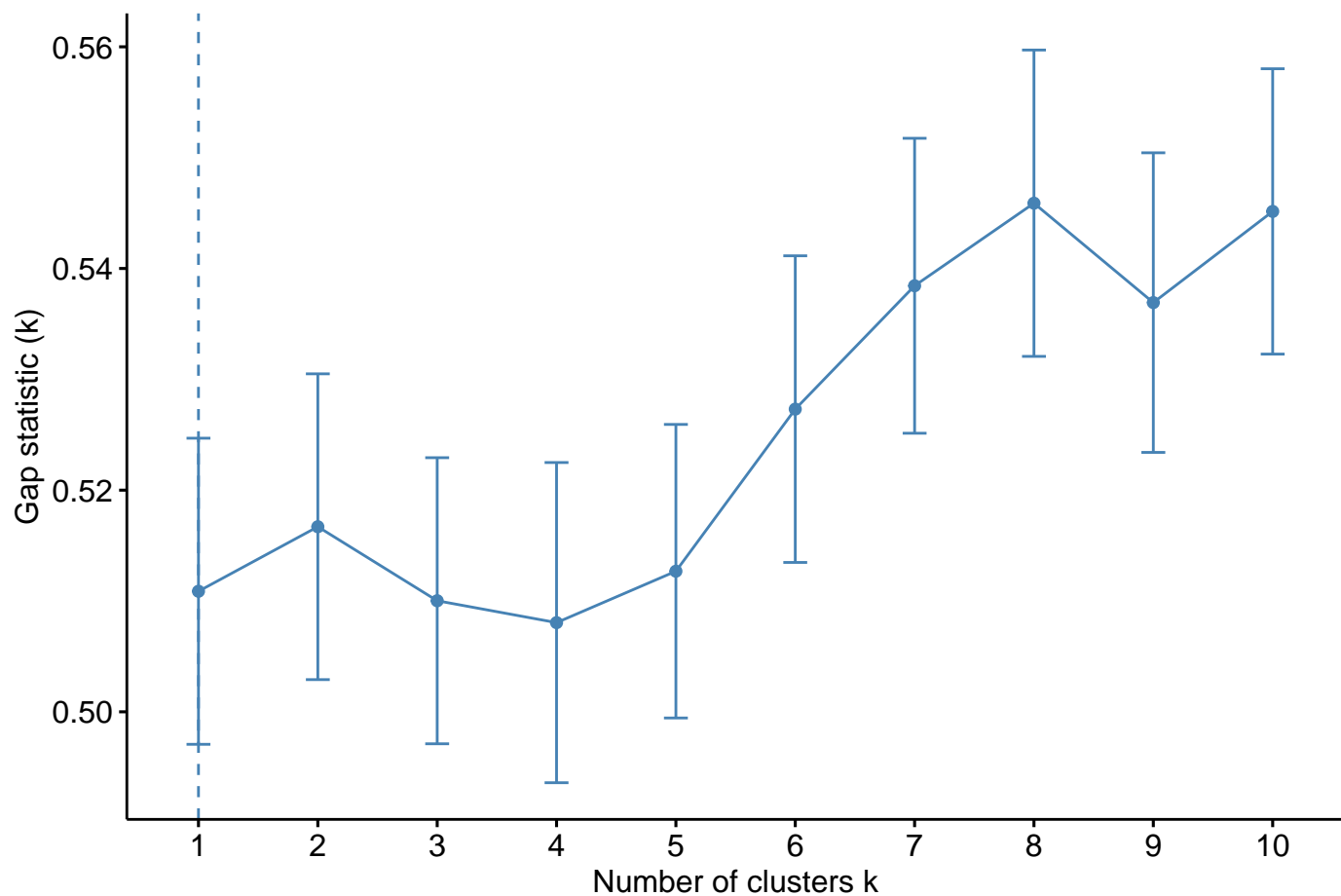

Supplement: Supplementary file 1 [file jcm-13-06824-s001.zip › Figure S5 - Gap statistic.pdf]
